# Tetramethylol acetylenediurea: MAK Value Documentation – Translation of the German version from 2023

**DOI:** 10.34865/mb539550kske10_3or

**Published:** 2025-09-29

**Authors:** Andrea Hartwig, MAK Commission

**Affiliations:** 1 Institute of Applied Biosciences. Department of Food Chemistry and Toxicology. Karlsruhe Institute of Technology (KIT) Adenauerring 20a, Building 50.41 76131 Karlsruhe Germany; 2 Permanent Senate Commission for the Investigation of Health Hazards of Chemical Compounds in the Work Area. Deutsche Forschungsgemeinschaft, Kennedyallee 40, 53175 Bonn, Germany. Further information: Permanent Senate Commission for the Investigation of Health Hazards of Chemical Compounds in the Work Area | DFG

**Keywords:** tetramethylol acetylenediurea, nose, larynx, irritation, formaldehyde releaser, MAK value, maximum workplace concentration, carcinogenicity, germ cell mutagenicity, developmental toxicity

## Abstract

The German Senate Commission for the Investigation of Health Hazards of Chemical Compounds in the Work Area (MAK Commission) has evaluated the data for tetramethylol acetylenediurea [5395-50-6] to derive an occupational exposure limit value (maximum concentration at the workplace, MAK value) considering all toxicological end points. Relevant studies were identified from a literature search and also unpublished study reports were used. Tetramethylol acetylenediurea releases formaldehyde in aqueous solution. Tetramethylol acetylenediurea is expected to occur in aerosol form because it has a very low vapour pressure. At a concentration of 21.7 mg/m^3^ slight irritation in the larynx of rats occurs probably due to the impaction of the aerosol used in the study. Effects in the nasal cavity were observed at 96 mg/m^3^ and above. Since 2014, the Commission has used an empirical approach for deriving MAK values for substances that induce effects on the upper respiratory tract as their critical effect. On this basis, the maximum concentration at the workplace (MAK value) has been set at 0.5 mg/m^3^. As the critical effect of tetramethylol acetylenediurea is irritation, Peak Limitation Category I has been assigned with an excursion factor of 2 in analogy to the classification made for formaldehyde. In rats, the NOAEL for developmental toxicity induced by tetramethylol acetylenediurea is 450 mg/kg body weight and the NOAEL for perinatal and parental toxicity is 1000 mg/kg body weight. As no teratogenicity was observed and the margins between the NOAELs and the MAK value are sufficiently large, tetramethylol acetylenediurea has been assigned to Pregnancy Risk Group C. Formaldehyde was classified in Carcinogen Category 4 because it causes nasal tumours at concentrations that exceed the detoxification capacity of that tissue. Studies investigating the carcinogenicity and germ cell mutagenicity of tetramethylol acetylenediurea are not available. Thus, in analogy to the classifications made for formaldehyde, the substance has been assigned to Carcinogen Category 4 and Germ Cell Mutagenicity Category 5. Clinical findings show sensitizing effects on the skin and tetramethylol acetylenediurea has therefore been designated with “Sh”. There are no data for respiratory sensitization. Dermal absorption is not expected to contribute significantly to systemic toxicity.

**Table:** 

**MAK value (2022)**	**0.5 mg/m^3^ I (inhalable fraction)**
**Peak limitation (2022)**	**Category I, excursion factor 2**
	
**Absorption through the skin**	**–**
**Sensitization (2022)**	**Sh**
**Carcinogenicity (2022)**	**Category 4**
**Prenatal toxicity (2022)**	**Pregnancy Risk Group C**
**Germ cell mutagenicity (2022)**	**Category 5**
	
**BAT value**	**–**
	
Synonyms	1,3,4,6-tetra(hydroxymethyl)-[3aH,6aH]-1,3,4,6-tetraazabicyclooctan-2,5-dione 1,3,4,6-tetrakis(hydroxymethyl)tetrahydroimidazo[4,5-d]imidazole-2,5(1H,3H)-dione tetrahydro-1,3,4,6-tetrakis(hydroxymethyl)imidazo[4,5-d]imidazole-2,5(1H,3H)-dione tetramethylolglycoluril
Chemical name (IUPAC)	1,3,4,6-tetrakis(hydroxymethyl)-3*a*,6*a*-dihydroimidazo[4,5-d]imidazole-2,5-dione
CAS number	5395-50-6
Structural formula	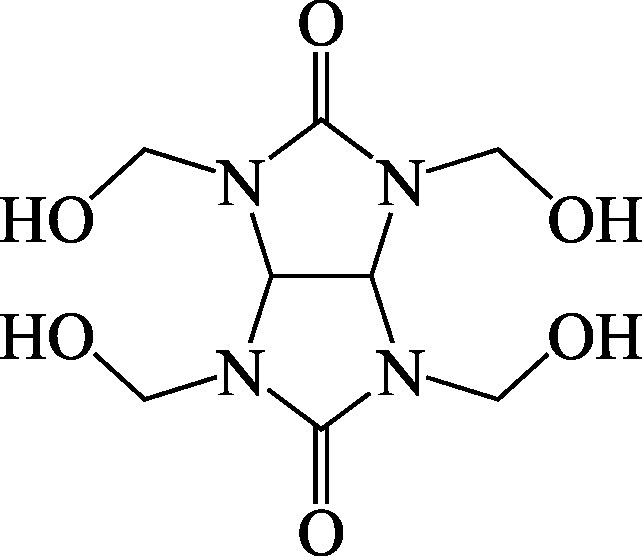
Molecular formula	C_8_H_14_N_4_O_6_
Molar mass	262.22 g/mol
Melting point	–13 °C (ECHA 2018) –22 °C according to OECD Test Guideline 102 (BASF AG 2002 a)
Boiling point at 1013.25 hPa	100.2 °C (ECHA 2018)
Density at 20 °C	1.215 g/cm^3^ (ECHA 2018)
Vapour pressure at 25 °C Vapour pressure at 20 °C	7.6 × 10^–10 ^hPa (calculated, US EPA 2022) 20.4–23.3 hPa, determined according to OECD Test Guideline 104, 50% aqueous solution (BASF AG 2002 f, see also Section 3.1.1)
log K_OW_	–2.0 (ECHA 2018)
Solubility	completely soluble in water (no other details; ECHA 2018)
	
Hydrolytic stability	releases formaldehyde during hydrolysis, rate of hydrolysis dependent on pH (Section 3.1.2; Dr. U. Noack-Laboratorium für angewandte Biologie 2003 a)
Stability	thermally stable (ECHA 2018)
Production	addition of 4 moles of formaldehyde to 1 mole of acetylenediurea (Paulus 2004)
Purity	97%–100% (ECHA 2018)
Impurities	–
Uses	biocide, product protection; antimicrobial effect in metal-working fluids, household and cosmetic products, textiles, fillers, pharmaceuticals (NICNAS 2016), not authorized as an ingredient in cosmetic products in the EU (European Parliament and European Council 2009)
Concentrations used	product 1: 0.013%–0.2% for various products 0.13% for metal-working fluids product 2: 0.025%–1.5% for various products 0.025%–0.075% for metal-working fluids (CTGB 2013)

Note: releases formaldehyde

Tetramethylol acetylenediurea (TMAD) is a formaldehyde releaser with an average ratio of formaldehyde (CAS number: 50-00-0) to acetylenediurea (CAS number: 496-46-8) of 3.1:1 to 3.5:1 (BfR 2007). Cited unpublished toxicological studies from companies have been made available to the Commission.

## 1 Toxic Effects and Mode of Action

Tetramethylol acetylenediurea releases formaldehyde. Hydrolysis takes place with a half-life of < 1 hour in the neutral range and rapidly in the alkaline range. Even under acidic conditions (pH 4), TMAD is hydrolytically unstable in a 0.01% solution with a half-life of about 90 days. Formaldehyde is carcinogenic in the nose of rats when exposed by inhalation.

After exposure for 28 days, TMAD caused squamous cell metaplasia in the larynx at a concentration of 21.7 mg/m^3^ and above in female and male Wistar rats and hyperplasia in 1 female animal. At concentrations of 96 mg/m^3^ and above, hyperplasia occurred also in the nasal cavity of male and female animals.

Tetramethylol acetylenediurea is slightly irritating to the skin and not irritating to the eye.

There are reports of contact sensitization in humans after exposure to TMAD. Positive results in a maximization test in guinea pigs likewise provide evidence of a sensitization potential. There are no findings of respiratory sensitization.

Tetramethylol acetylenediurea was clastogenic in the chromosomal aberration test in V79 cells; this has not been confirmed in vivo.

In a prenatal developmental toxicity study with gavage administration, doses of and above 405 mg/kg body weight and day led to increased mortality and reduced body weights in the dams. Foetal weights were reduced at 1215 mg/kg body weight and day.

## 2 Mechanism of Action

The biocidal effect occurs via the release of formaldehyde in aqueous solutions. The quantity of formaldehyde released depends on the amount of TMAD, the proportion of water in the product, the pH and the storage time and temperature (NICNAS 2016).

Formaldehyde has strong irritant and carcinogenic effects after inhalation exposure (Greim 2002). It can be assumed that formaldehyde is released on contact with the mucous membranes of the respiratory tract due to spontaneous hydrolysis of TMAD.

The sensitizing effect of TMAD is due to formaldehyde (Hartwig 2014; NICNAS 2016).

The hydrolysis product acetylenediurea cannot be responsible for the effects observed with TMAD, as the substance was not irritating to the eyes and skin and was not toxic after oral doses of up to 1000 mg/kg body weight and day in a 28-day study (ECHA 2019). Acetylenediurea was also not genotoxic in an in vivo micronucleus test in male mice and not mutagenic in a test with the Salmonella strain TA100 (NTP 2018 a, b).

There are no data available for the other hydrolysis products trimethylol acetylenediurea, dimethylol acetylenediurea and monomethylol acetylenediurea.

## 3 Toxicokinetics and Metabolism

### 3.1 Absorption, distribution, elimination

After single gavage doses of TMAD (not radiolabelled) of 200 or 1000 mg/kg body weight, the amount absorbed in rats (strain and sex not specified) was 22%. The faeces contained 78% of the administered dose. In the following 7 days, 2%–9% of the administered dose was excreted with the urine. In the blood, a value of 0.2% was not exceeded; the maximum concentration in the blood was reached after 2 hours (no other details; CTGB 2013; NICNAS 2016).

After semi-occlusive application of 100 µl of a 3% aqueous solution of TMAD with ^14^C-labelled carbonyl groups to the skin (exposed area 9.6 cm^2^ for 6 hours) of 15 male Wistar rats according to OECD Test Guideline 427, 87% was not absorbed. After 6 hours, 1%–4% of the applied radioactivity was found in the stratum corneum and 0.4%–3% in the deeper skin layers. After 48 hours, the amount excreted in the faeces was 0.04% and that in the urine 0.3%. The radioactivity in the blood, organs and tissues was negligibly low 6 hours after administration and was no longer detectable after 48 hours (Fraunhofer ITEM 2008).

Based on the in vivo test, the expected systemic exposure resulting from dermal absorption under standard conditions can be calculated as follows: 100 µl of a 3% TMAD solution (v/v) contains 4.02 mg TMAD (3 µl × density of 1.34 mg/µl). Of this, 13.7 µg TMAD is absorbed systemically (excretion of 0.34% with the faeces and urine). For an exposed area of 9.6 cm^2^ and an exposure duration of 6 hours, this results in a flux of 0.237 µg/cm^2^ and hour (13.7 µg/9.6 cm^2^/6 hours). For exposure of 2000 cm^2^ of skin for 1 hour (standard conditions for dermal exposure), this results in a systemically absorbed amount of 0.475 mg TMAD.

#### 3.1.1 Vapour pressure

For substances with a vapour pressure of less than 1 hPa and more than 10^–5^ hPa at room temperature, sampling methods that capture vapour and aerosol simultaneously in one sampling system should generally be selected (DFG 2022). Substances with a vapour pressure of less than 10^–5^ hPa are present as an aerosol when inhaled.

The hydrolysis of a formaldehyde releaser is intensified by the increasing dilution in the aqueous environment of the respiratory tract depending on the substance-specific hydrolysis rate. This applies equally to vapour and aerosols. However, the presence of aerosol particles can lead to a higher (localized) concentration on the mucous membranes of the respiratory tract (aerosol impaction). The formaldehyde releaser can therefore be more irritating to the respiratory tract than the vaporous formaldehyde itself.

The problem can be illustrated using *N*,*N*′,*N*′′-tris(β-hydroxyethyl)hexahydro-1,3,5-triazine as an example. The local effects in the lungs after exposure to 3 mg/m^3^ are stronger than those of its hydrolysis products formaldehyde and 2-aminoethanol. This is presumably the result of aerosol impaction. The calculated vapour pressure of *N*,*N*′,*N*′′-tris(β-hydroxyethyl)hexahydro-1,3,5-triazine is 5 × 10^–8^ hPa. Formaldehyde and 2-aminoethanol themselves would theoretically both be present in vapour form at the LOAEC (lowest observed adverse effect concentration) of *N*,*N*′,*N*′′-tris(β-hydroxyethyl)hexahydro-1,3,5-triazine (3 mg/m^3^) obtained in this inhalation study. The unexpectedly strong effects here can therefore be explained by a local increase in formaldehyde and aminoethanol concentrations resulting from hydrolysis after the aerosols come into contact with the aqueous environment of the respiratory tract (DFG 2022; Hartwig and MAK Commission 2025).

After inhalation exposure to TMAD aerosols (in aqueous dilution), effects in the larynx were observed at lower concentrations than those at which effects on the respiratory epithelium of the nose were found. This result indicates that also here the aerosols of TMAD had a stronger effect than the hydrolysis product formaldehyde, which in the vapour state only leads to histopathological changes in the nose (TNO 2005).

Specification of the vapour pressure of the undiluted substance is therefore necessary for the interpretation of inhalation studies. The reported vapour pressure of 23.3 hPa for TMAD at 20 °C (ECHA 2018) corresponds to the vapour pressure of water of 23.38 hPa at 20 °C, as an aqueous TMAD solution was measured (BASF AG 2002 f).

The calculated vapour pressure of TMAD is 7.6 × 10^–10^ hPa (US EPA 2022).

There is no study available in which the vapour pressure for undiluted TMAD is given.

#### 3.1.2 Hydrolysis

Tetramethylol acetylenediurea contains 46% formaldehyde (NICNAS 2016). A maximum of 23% formaldehyde can be released from an aqueous solution containing 50% TMAD (w/w) (JRF 2001, 2002).

After passing air over the substance (room temperature), a total of 1 mg formaldehyde was released from 1 g TMAD (Gewerbehygiene I.G. Labor 1943).

An aqueous TMAD solution of approximately 50% contains about 3% free formaldehyde (BASF AG 1979).

The dependence of the hydrolysis of TMAD on the pH was investigated according to OECD Test Guideline 111. In buffer solutions with the pH values 4, 7 and 9, 100 mg TMAD/l was dissolved at 20 °C or 30 °C. The test at pH 4 was repeated. The decrease in the TMAD concentration was determined by HPLC and UV detection at 196 nm. The half-lives at pH 4 were 95 and 83 days at a temperature of 20 °C, and 15.9 and 14.6 days at 30 °C. At pH 7, the half-lives were 0.15 days (3.55 hours) at 20 °C and 0.04 days (0.9 hours) at 30 °C. In alkaline solution (pH 9), complete hydrolysis took place spontaneously (Table 1). The hydrolysis products were trimethylol acetylenediurea, dimethylol acetylenediurea, monomethylol acetylenediurea, acetylenediurea and formaldehyde (Dr. U. Noack-Laboratorium für angewandte Biologie 2003 a).

**Tab. 1:** Reaction rate constants and half-lives of 0.01% TMAD at different pH values and temperatures (Dr. U. Noack-Laboratorium für angewandte Biologie 2003 a)

**Parameter**	**pH 4 (I)**		**pH 4 (II)**		**pH 7**		**pH 9**	
	**20 °C**	**30 °C**	**20 °C**	**30 °C**	**20 °C**	**30 °C**	**20 °C**	**30 °C**
k_obs_ [1/s]	8.44 × 10^–8^	5.04 × 10^–7^	9.66 × 10^–8^	5.48 × 10^–7^	5.42 × 10^–5^	2.13 × 10^–4^	–^a)^	–^a)^
half-life [h]	2279.7	381.9	1992.8	351.2	3.55	0.90	–^a)^	–^a)^
half-life [d]	95.0	15.9	83.0	14.6	0.15	0.04	–^a)^	–^a)^

^a)^ spontaneous complete hydrolysis

k_obs_: reaction rate constant

The hydrolysis of TMAD at pH 1.2 and 37 °C was investigated according to OECD Test Guideline 111. 100 mg TMAD/l was dissolved in the buffer solution. The decrease in the TMAD concentration was determined using HPLC and UV detection at 196 nm. The half-life at pH 1.2 was 1.24 days (29.79 hours). Determination of the reaction rate yielded constants of 6.46 × 10^–6^/s and 2.33 × 10^–2^/h. Hydrolysis products were trimethylol acetylenediurea, dimethylol acetylenediurea, monomethylol acetylenediurea, acetylenediurea and formaldehyde (Dr. U. Noack-Laboratorium für angewandte Biologie 2003 b).

#### 3.1.3 Summary

Tetramethylol acetylenediurea releases formaldehyde. Hydrolysis is rapid at pH 7 (half-life < 1 hour at 30 °C), but slow in the acidic range. In alkaline solution (pH 9), complete hydrolysis takes place spontaneously. Hydrolysis products are trimethylol acetylenediurea, dimethylol acetylenediurea, monomethylol acetylenediurea, acetylenediurea and formaldehyde.

### 3.2 Metabolism

There are no data available.

## 4 Effects in Humans

### 4.1 Single exposures

There are no data available.

### 4.2 Repeated exposure

There are no data available.

### 4.3 Local effects on skin and mucous membranes

In 6 test persons, tetramethylol acetylenediurea did not have irritant effects 24 hours after direct application of the substance moistened with water or oil or after the application for 72 hours of a solution of 10 g TMAD with 0.5 g ammonium nitrate in 100 ml water (no other details; Gewerbehygiene I.G. Labor 1943).

### 4.4 Allergenic effects

#### 4.4.1 Sensitizing effects on the skin

A total of 7 studies with epicutaneous testing of TMAD are available (see Table 2). Tetramethylol acetylenediurea was obtainable as a 5% aqueous patch test preparation until 1999 (de Groot et al. 2010).

In one study, 11 of 13 patients with suspected contact dermatitis caused by textiles reacted to one or more formaldehyde-releasing substances (6 individual substances, including formaldehyde, as well as 1,3-ethyleneurea (2-imidazolidinone) and a melamine-formaldehyde mix) in the patch test, one of which yielded a positive result for TMAD (Sherertz 1992).

**Tab. 2:** Reports of patch test reactions to TMAD in patients with suspected contact allergy

**Tested persons**	**Concentration, vehicle**	**Results:** **reaction in**	**Comments**	**References**
13	5% aqueous	1 of 13	**Test period**: 1988–1991 **Collective**: From a total collective of 462 patients, 13 (9 ♀, 4 ♂) with suspected allergic contact dermatitis caused by clothing were tested with TMAD. The positive reaction was confirmed in a second test. **Reading time**: D2, D3	Fowler et al. 1992; Sherertz 1992
17	5% aqueous	8 of 17	**Test period**: 1988–1990 **Collective**: From a total collective of 1022 patients from 2 clinics (636 ♀, 386 ♂, average age 44 years) with dermatitis of unknown origin, 17 patients were tested with TMAD, of which 8 reacted to TMAD and 16 reacted to formaldehyde-based dyes and resins. **Reading time**: D2, D4 or D7	Fowler et al. 1992
10	5% aqueous	8 of 10	**Test period**: probably 1994–1997 **Collective**: From a total collective of 12 patients, 10 (9 ♀, 1 ♂ between 24 and 77 years of age), who had previously reacted to formaldehyde resins, were tested with TMAD. Formaldehyde and other substances for textile treatment were also tested (including dimethylol dihydroxyethylene urea, dimethylol propylene urea, melamine-urea-formaldehyde condensates) **Reaction strength**: 1 × 1+; 3 × 2+; 4 × 3+ **Reading time**: D3	Scheman et al. 1998
18	5% aqueous	2 of 18	**Test period**: 5 years (no other details) **Collective**: From a total collective of 1400 patients who were referred to a clinic within 5 years with suspected contact dermatitis, 18 (14 ♀, 4 ♂, between 15 and 62 years, average age 39 years) were diagnosed of having contact dermatitis caused by clothing. Two patients reacted to TMAD: a 41-year-old female patient with generalized symptoms, but no clear correlation with textile intolerance; a 42-year-old female patient with dermatitis in the chest, trunk, abdomen and groin area; positive reactions to formaldehyde and other substances in both patients **Reading time**: D2–D3	Fuentes Cuesta et al. 2000
9	5% aqueous	1 of 9	**Test period**: 1998 **Collective**: Of a total of 103 patients with suspected contact dermatitis in connection with textile constituents, 9 hospital patients with purpuric allergic contact dermatitis to textile constituents (patchy skin changes) were tested with TMAD. 30 of the 103 patients reacted to an allergen of the series. Further details of the reaction to TMAD in 21 of these patients are not available. **Reading time**: D2, D3, D7	Lazarov and Cordoba 2000
286	5% aqueous	6 of 286	**Test period**: within 1 year (no other details) **Collective**: 286 patients (190 ♀, 96 ♂, average age 48 years) with suspected allergic contact dermatitis **Reading time**: D2 and D3 probably collective overlap with Lazarov and Cordoba (2000)	Lazarov et al. 2002
188	5% aqueous	21 of 188	**Test period**: 1994–1999 **Collective**: patients who had allergic reactions to non-iron fabric treatments. Of the 21 people who tested positive for TMAD, 16 reacted also to formaldehyde **Reaction strength**: 1+ and 2+ **Reading time**: D2 and D4	Metzler-Brenckle and Rietschel 2002

D: day after application

In another study, it was observed that 16 of 678 patients with dermatitis of unknown origin were allergic to formaldehyde-based dyes and resins; 8 of these patients were tested with a positive result for TMAD in the patch test (Fowler et al. 1992). In both studies, most of the patients who reacted to TMAD also reacted to formaldehyde (Hatch and Maibach 1995).

In a study from 1997, 8 of 10 patients who had previously had an allergic reaction to a non-iron fabric treatment reacted to TMAD in a patch test (Scheman et al. 1998).

Of 1400 patients referred to a dermatological clinic within 1 year, 18 patients were suspected of having contact dermatitis caused by textiles. In the patch test, 2 of 18 persons reacted to TMAD (Fuentes Cuesta et al. 2000).

Furthermore, 2 studies by Lazarov et al. are available. In the first study (in 1998), 103 hospital patients suspected of having allergic contact dermatitis caused by clothing were tested for reactions to commercially available allergen preparations of the “Textile Colour & Finish Series (TCFS)” and a standard series. Thirty of the 103 patients reacted to allergens of the TCFS series, 9 of them demonstrated purpuric macules, papules and patches. Of these 9 patients, 1 reacted to TMAD in the patch test (Lazarov and Cordoba 2000).

In the second study, in which 286 patients underwent patch testing with the same textile and standard series within 1 year, 6 patients reacted to TMAD (Lazarov et al. 2002). There is probably a collective overlap with Lazarov and Cordoba (2000).

In the period from 1994 to 1999, 21 of 188 patients who had an allergic reaction to a non-iron fabric treatment of clothing fabrics reacted to TMAD in the patch test (Metzler-Brenckle and Rietschel 2002).

#### 4.4.2 Sensitizing effects on the airways

There are no data available.

### 4.5 Reproductive and developmental toxicity

There are no data available.

### 4.6 Genotoxicity

There are no data available.

### 4.7 Carcinogenicity

There are no data available.

## 5 Animal Experiments and in vitro Studies

### 5.1 Acute toxicity

#### 5.1.1 Inhalation

Exposure to saturated TMAD vapour for 7 hours did not result in mortality in 12 rats (no other details; BASF AG 1979, 2002 c).

#### 5.1.2 Oral administration

After oral administration of TMAD to groups of 5 female and 5 male Sprague Dawley rats, the LD_50_ was greater than 5000 mg/kg body weight. After doses of 2150 or 5000 mg/kg body weight, the animals were in a poor general condition with diarrhoea, apathy and spastic gait. In addition, dyspnoea and ruffled fur were observed in the animals of the high dose group and 2 males died after only 2 days (BASF AG 1979, 2002 e).

#### 5.1.3 Dermal application

The LD_50_ is greater than 2000 mg/kg body weight in rats (CTGB 2013).

#### 5.1.4 Intraperitoneal injection

The LD_50_ was greater than 2000 mg TMAD/kg body weight after intraperitoneal injection in groups of 5 male and 5 female NMRI mice. After the injection of 700 or 2000 mg/kg body weight, the animals were in a poor general condition with dyspnoea, apathy, spastic gait and ruffled fur. In addition, staggering was observed in the animals of the high dose group and 2 male animals died after 14 days (BASF AG 1979, 2002 d).

### 5.2 Subacute, subchronic and chronic toxicity

#### 5.2.1 Inhalation

In a preliminary study to estimate the exposure concentration for the subsequent subacute study, groups of 5 female and 5 male Wistar rats were exposed nose-only to concentrations of 0, 54, 120 or 259 mg TMAD/m^3^ (nominal concentrations 0, 45, 115 or 230 mg TMAD/m^3^) for 6 hours daily on 5 days. The concentration of the initial substance used was given as 58.7% TMAD in water (corresponding to 26.9% formaldehyde) and the vapour pressure of this solution as 20.4 hPa at 20 °C. To generate the TMAD concentrations used, 10.4 g of the initial substance was dissolved in 244 g of demineralized water. The aerosols of the concentrations used, generated with the aid of an air-driven nebulizer, had average diameters of 2.2, 1.8 and 2.1 µm. The test atmosphere consisted of vapour and aerosol. The TMAD concentration in the test atmosphere was checked as in the 28-day study (see below). The determination of the aerosol/vapour ratios in the exposure atmosphere for the 3 exposure groups yielded aerosol proportions of 75%, 65% and 71%. The remainder was present as vapour. The clinical examination and gross-pathological examination after the last exposure did not reveal any substance-related effects, nor were any effects on body weight development, food consumption or organ weights observed (TNO 2005).

In a 28-day inhalation study, groups of 10 male and 10 female Wistar rats (Crl:(WI)WU BR) were exposed nose-only to concentrations of 0, 21.7, 96 or 512.9 mg/m^3^ (target concentrations 0, 20, 100 or 500 mg TMAD/m^3^) for 6 hours daily on 5 days per week. The animals were examined the day after the last exposure. The concentration of the initial substance used was given as 58.7% TMAD in water (corresponding to 26.9% formaldehyde) and the vapour pressure of this solution as 20.4 hPa at 20 °C. To generate the TMAD concentrations used, 15 g of the initial substance was dissolved in 150 g of demineralized water. The respective exposure concentration was adjusted by dilution with air. The aerosols generated by atomization had a diameter of 1–3 µm (mean mass median diameter (MMAD): 2.4, 2.0, 2.7 µm). The test atmosphere consisted of vapour and aerosol. Samples were transferred to dilute hydrochloric acid (0.25 l/min) to regularly check the TMAD concentration of the different test atmospheres, as TMAD hydrolyses very slowly in the acidic pH range. To determine the total amount of formaldehyde in the respective test atmosphere, NaOH was added to the solution, as hydrolysis to formaldehyde is already complete at pH 9, and HPLC was carried out (formaldehyde determined by measuring the luterine derivative). The formaldehyde concentrations determined in the test atmosphere were 0, 22 ± 1, 96 ± 6 and 512 ± 23 mg/m^3^, respectively. In addition, a gravimetric determination was carried out to measure the aerosol quantity as an increase in filter weight. This yielded values of 0, 16 ± 1, 79 ± 4 and 398 ± 12 mg/m^3^, respectively. From the ratio between the gravimetric determination and the total formaldehyde quantity, it can be concluded that 76%, 82% and 78% of the 3 exposure atmospheres were present as aerosol. The observed effects are listed in Table 3. Even at the lowest concentration, squamous cell metaplasia occurred in the larynx, predominantly in the epiglottis, the severity of which increased with the concentration and then manifested itself as hyperplasia. One female animal exhibited hyperplasia of the larynx even at the lowest concentration. In the middle and high concentration groups, hyperplasia was observed in section levels 2 and 3 of the respiratory epithelium of the nose. No effects were observed in the pharynx and lungs. Neurobehavioural or motor disorders were not observed. The examination of the eyes, body weight development and food intake did not yield unusual findings. Minor changes in haematological parameters were within the range of the historical control data. In the authors’ opinion, apart from the effects on the larynx and nose and the decrease in relative thymus weights in the high concentration group, there were no substance-related effects, as all other deviations were not concentration-dependent or were within the range of the historical controls (TNO 2005). The determined vapour pressure of 20.4 hPa (20 °C) differed considerably from the calculated vapour pressure of 7.6 × 10^–10^ hPa. Presumably the vapour pressure of the water was measured.

**Tab. 3:** Toxicity of TMAD after repeated inhalation exposure (TNO 2005)

**Species,** **strain,** **number per group**	**Exposure**	**Findings**
rat, Wistar, 5 ♂, 5 ♀	5 days, 0, 54, 120, 259 mg/m^3^ 6 hours/day, 5 days/week, MMAD 1–3 µm, 2.5% in water, nose-only	**259 mg/m^3^**: **NOAEC**
rat, Wistar, 10 ♂, 10 ♀	28 days, 0, 21.7, 96, 512 mg/m^3^ 6 hours/day, 5 days/week, MMAD 2.0–2.8 µm, 5.87% in water, nose-only	**21.7 mg/m**^3^: **LOAEC** kidneys: ♀: absolute and relative weights ↑; **21.7 mg/m**^3^**and above**: blood: ♂: AP ↓ (not at 512 mg/m^3^), ♀: phosphate ↑ (determined in plasma), larynx: squamous cell metaplasia (very slight ♂: 6/10, ♀: 7/10), hyperplasia (♀: 1/10); **96 mg/m^3^**: thyroid gland: ♀: absolute and relative weights ↓, adrenal glands: relative weights ↓; **96 mg/m^3^ and above**: blood: ♀: albumin ↓ (2%), nose: hyperplasia in the respiratory epithelium (very slight, section level 2, 3 and 4), ♂: squamous cell metaplasia (very slight, section level 3, not statistically significant), larynx: squamous cell metaplasia (very slight to moderate ♂: 9/10, ♀: 7/10), squamous cell hyperplasia (♂: 5/9, ♀: 6/7), parakeratosis and scaling (♀: 3/10), hyperplasia in the respiratory epithelium (slight ♀: 2/10); **512 mg/m^3^**: ♀: sparse fur (4/10), thymus: ♀: relative weights ↓, blood: ♀: albumin ↓ (5%), ♂: Ca^2+^ ↑ (plasma) nose: squamous cell metaplasia (slight to severe, transitional and respiratory epithelium, section level 1–5), infiltration of mononuclear cells (section level 2–4), larynx: squamous cell metaplasia (slight to moderate ♂: 8/10, ♀: 10/10), squamous cell hyperplasia (♂: 8/8, ♀: 10/10), ulcerations (4/20), infiltration of inflammatory cells (♂: 4/10, ♀: 8/10), ♂: hyperplastic changes in the respiratory epithelium, ♀: microcysts (2/10), trachea/bronchi: hyperplastic changes at the bifurcation (♂: 3/10)

AP: alkaline phosphatase; LOAEC: lowest observed adverse effect concentration; NOAEC: no observed adverse effect concentration

Squamous metaplasia of the larynx occurring in the epiglottis can be considered not to be adverse if the severity is low. In the larynx, unlike the respiratory epithelium, hyperplasia develops from the squamous cell metaplasia, which represents an intensification of the effect (Kaufmann et al. 2009).

#### 5.2.2 Oral administration

In a gavage study carried out according to OECD Test Guideline 407, groups of 5 male and 5 female Wistar rats were given TMAD doses of 0, 100, 300 or 1000 mg/kg body weight and day for 28 days and 5 additional animals per dose for a 14-day follow-up period. Three male and 3 female Wistar rats were given 0 or 1000 mg/kg body weight and day over a period of 14 days to test the irritant effect on the gastrointestinal tract. The TMAD was dissolved in distilled water. The solution was prepared daily and its stability was determined by measuring formaldehyde. Haematological examination revealed a statistically significant increase in the number of monocytes at 300 mg/kg body weight and above; this effect was no longer visible after the 14-day recovery period. A statistically significant increase in the mean corpuscular volume (MCV, p < 0.05) in all treated females was accompanied by a statistically significant decrease in the mean corpuscular haemoglobin concentration (MCHC, p < 0.01) in the high dose group, but had no effect on the red blood cell count and haemoglobin content. Aspartate aminotransferase activity was significantly reduced in the high dose group and returned to normal after 14 days of recovery. All treated female animals had marginally increased protein levels and slightly but significantly increased phosphate levels at and above 300 mg/kg body weight and day, which were not accompanied by histopathological renal effects. After the 14-day recovery period, the relative liver weights were increased by about 17% only in the high-dose female animals. Organ weight changes in the prostate, spleen and liver were regarded by the authors as not substance-related, as they were not accompanied by histopathological changes and did not occur in the recovery group. Histopathological examination of the liver, heart, kidneys and spleen of all animals did not reveal any abnormalities. No clinical signs, increased mortality, body weight reduction, changes in the eyes or behavioural abnormalities were observed. In the opinion of the authors, the NOAEL (no observed adverse effect level) in this study was the highest dose tested of 1000 mg/kg body weight (ECHA 2018; JRF 2001).

In a 2-generation study carried out according to OECD Test Guideline 416, groups of 24 male and 24 female Wistar rats (Crl: (WI) BR) were given TMAD doses of 0, 100, 300 or 1000 mg/kg body weight and day by gavage (7 days per week, vehicle: distilled water) for 10 weeks before mating, during mating and the females until day 21 of lactation. In female F0 animals, increased absolute and relative liver weights were observed at and above 300 mg/kg body weight and day (11% to 17%), which were attributed to the very low liver weights of the control animals in this study. In both generations, there were no conspicuous changes in the parameters mortality, clinical signs, body weights, food consumption, gross pathological and microscopic examinations, organ weights, sperm parameters and oestrus cycle (see also Section 5.5.1; NOTOX B.V. 2007 a). The NOAEL for parental toxicity was 1000 mg/kg body weight and day.

In accordance with OECD Test Guideline 408, groups of 10 male and 10 female Wistar rats were given TMAD doses of 0, 63, 250 or 1000 mg/kg body weight daily for 90 days by gavage. Ten additional male and female control and high dose animals were included for a follow-up observation period of 28 days. Haematological examination revealed a statistically significant increase in MCV and mean corpuscular haemoglobin in the male animals of the high dose group, but no increase in red blood cell (RBC) counts, haemoglobin levels or MCHC. There was also a statistically significant increase in the monocyte count in the high dose group male animals, which was, however, within the range of historical control data. In the female animals of all dose groups, statistically significant increases in the red blood count, and haemoglobin and haematocrit values were observed. However, the changes were not dose-dependent and the values were within historical control data. In the high dose groups, prolonged clotting time was observed in male rats and decreased clotting time in females at the end of the recovery period. Reduced γ-glutamyltranspeptidase (γ-GTP) activity occurred only at the end of the recovery period in the male animals of the high dose group. Significant changes in clinico-chemical parameters in the female animals (increased bilirubin and chloride) were within the range of the historical control data. The relative organ weights were unaffected, except for increased relative brain weights in the males of the high dose group, which was within the range of the historical control data. The animals in the high dose group exhibited lesions in the liver and brain (females) and hyperplasia in the small intestine (males) more frequently than the control animals. No other clinical signs, nor increased mortality, body weight reduction, changes in the eyes, effects on sperm or behavioural abnormalities (functional observational battery) were observed. The effects that occurred were classified by the authors as not substance-related. All animals, including the controls, had a mycoplasma infection in the lungs, which in the authors’ opinion led to hyperplasia in the spleen and lymphatic vessels, but did not affect the other end points. In the opinion of the authors, the NOAEL in this study was the highest dose tested of 1000 mg/kg body weight (ECHA 2018; JRF 2002). The stability of the administered TMAD solution had been examined and confirmed in the 28-day study (see above) and was not checked again.

The effects in the small intestine in the 90-day study were evaluated by other institutions as a substance-related irritant effect (formaldehyde); a NOAEL of 250 mg TMAD/kg body weight was derived from this study (CTGB 2013).

A 28-day study with oral administration of the TMAD degradation product **acetylenediurea** (1000 mg/kg body weight and day) in Wistar rats likewise revealed haematological effects in the female animals. Small decreases in the haemoglobin and haematocrit levels that were not statistically significant were observed. A NOAEL of 1000 mg/kg body weight and day was derived from this study (ECHA 2019). The haematological effects observed with TMAD could therefore be attributed to acetylenediurea.

In a developmental toxicity study, which is not available in the original, according to the authors of the REACH registration dossier, oral TMAD doses of 0, 125, 405 or 1215 mg/kg body weight and day administered to rabbits resulted in the death of 2 animals each in the middle and high dose groups and reduced body weights. The NOAEL for maternal toxicity is thus 125 mg/kg body weight (no other details; ECHA 2018; NICNAS 2016).

**Conclusion:** In the studies with oral administration, minor effects were found in the haematological examination of the high dose groups. These were within the range of the historical control data, were not dose-dependent and there were no subsequent effects. Slight organ weight changes were not accompanied by histopathological effects. Therefore, the observed effects are not considered to be substance-related.

#### 5.2.3 Dermal application

After 28-day occlusive application of 0, 100, 300 or 600 mg TMAD/kg body weight and day (6 hours/day, 7 days/week) to the skin of 10 male and 10 female Wistar rats per group, all treated animals exhibited severe local irritation in the form of ulcerative dermatitis. The other effects observed were increased haematopoiesis, myeloid hyperplasia of the liver, spleen and bone marrow as well as enlarged axillary lymph nodes. These are considered by the authors to be the consequence of the severe dermatitis, as are clinical observations of restlessness, spasms, hunched posture, diarrhoea and laboured breathing, and not regarded as substance-related. The relative spleen weight was significantly increased in female rats at 100 mg/kg body weight and above. The high number of deaths of 11 of 20 animals in the high dose group and 8 of 20 in the middle dose group as well as the sacrifice of most animals in extremis demonstrate the high dermal toxicity of the substance. The weight losses that occurred in all groups cannot be assessed, as they were observed also in the control animals. Hearing ability, pupillary reflex, stating righting reflex and grip strength were examined to assess neurological behavioural or motor disorders, but like the ophthalmological examination did not yield any findings (no other details; NOTOX B.V. 2007 b).

### 5.3 Local effects on skin and mucous membranes

#### 5.3.1 Skin

After a 24-hour occlusive application of 0.5 ml of the undiluted substance to the intact or abraded skin of 6 albino rabbits, readings of the effects were carried out after 24 hours, 72 hours and after 7 days. Erythema with scores of 1.5 and 2.4 (on a scale with a maximum score of 4) and slight oedema with scores of 0.17 and 0.67 (of a maximum score of 4) were observed on the intact and abraded skin, respectively, after 24 and 72 hours, which were reversible within 7 days. Seven days after application, scabs were observed in all animals with abraded skin and in 3 animals with intact skin. The substance is considered slightly irritating according to the following evaluation of the irritation index on intact skin with a total value of 0.83 ((scores for erythema after 24 and 72 hours) + (scores for oedema after 24 and 72 hours)/N = 20/24): 0–0.5 not irritating, 0.6–3.0 slightly irritating, 3.1–5.0 moderately irritating and 5.1–8.0 severely irritating (BASF AG 1979; INBIFO 2002 b).

According to GHS criteria, the substance is regarded as not irritating to the skin (ECHA 2018).

After the application of 100 mg TMAD/kg body weight and day to the skin of rats for 28 days, all treated animals exhibited severe local irritation (ulcerative dermatitis) (no other details; Section 5.2.3; CTGB 2013). The original study is not available.

#### 5.3.2 Eyes

Undiluted TMAD (0.1 ml) was instilled into the conjunctival sac of one eye of 6 albino rabbits. The untreated eye of the animals served as the control. One hour after administration, all animals exhibited slight to moderate redness and slight to severe swelling of the conjunctiva, which were reversible after 24 hours. The substance was therefore assessed by the authors as not irritating (BASF AG 1979; ECHA 2018). A reconsideration of the data yielded an irritation score according to Draize of 3.22 (on a scale with a maximum score of 110; 0–10 not irritating, 11–25 slightly irritating, 26–56 moderately irritating and 57–110 severely irritating) (INBIFO 2002 a).

According to GHS criteria, the substance is regarded as not irritating to the eyes (ECHA 2018).

### 5.4 Allergenic effects

#### 5.4.1 Sensitizing effects on the skin

A maximization test was performed in 10 female guinea pigs (Dunkin Hartley) in accordance with OECD Test Guideline 406. Induction was carried out by intradermal application of a 5% solution of the test substance (49.5% w/w aqueous solution of TMAD) in 0.9% aqueous NaCl solution, followed by occlusive epicutaneous treatment. The lowest concentration causing irritation was the undiluted test substance, the highest non-irritant concentration was a 75% solution. However, due to the sticky consistency of the undiluted test substance, mechanical epilation was observed at the edge of the application area. Epicutaneous induction was therefore performed with 1 ml of a 75% aqueous solution (distilled water) on day 7. Fourteen days later, treatment was repeated with 0.5 ml of the 75% test substance. After 24 hours all 10 and after 48 hours 9 of 10 animals showed positive reactions (BASF AG 2002 b). The test result can therefore be regarded as positive.

#### 5.4.2 Sensitizing effects on the airways

There are no data available.

### 5.5 Reproductive and developmental toxicity

#### 5.5.1 Fertility

In a 2-generation study carried out according to OECD Test Guideline 416, groups of 24 male and 24 female Wistar rats (Crl: (WI) BR) were treated with 0, 100, 300 or 1000 mg TMAD/kg body weight and day by gavage (7 days/week, vehicle: distilled water) for 10 weeks before mating, during mating and the females until day 21 of lactation. In female F0 animals, increased absolute and relative liver weights were observed at and above 300 mg/kg body weight and day (11% to 17%), which were attributed to the low control values. In both generations, there were no unusual findings as regards mortality, clinical signs, body weights, food consumption, gross and histopathological examinations, organ weights, sperm parameters, oestrus cycle, fertility and reproductive parameters and the development of the offspring. The NOAEL for parental toxicity and fertility is 1000 mg TMAD/kg body weight and day (see also Section 5.2.2; NOTOX B.V. 2007 a).

#### 5.5.2 Developmental toxicity

In the range-finding study for the subsequent developmental toxicity study, a total of 16 pregnant New Zealand White rabbits were treated by gavage with 0, 500, 1250 or 3125 mg TMAD/kg body weight and day from days 5 to 29 of gestation and examined on day 30 of gestation. Prostration, diarrhoea and abdominal breathing were observed at 3125 mg/kg body weight and day. In addition, there were 2 deaths and 1 moribund animal at this dose, which is why the dose was reduced to 2000 mg/kg body weight and day. No toxicity was observed up to 1250 mg/kg body weight and day. Therefore, the doses for the subsequent developmental toxicity study were set at 0, 135, 405 and 1215 mg/kg body weight and day (JRF 2005).

In a prenatal developmental toxicity study carried out according to OECD Test Guideline 414, groups of 24 pregnant New Zealand White rabbits were treated by gavage with 0, 135, 405 or 1215 mg TMAD/kg body weight and day (vehicle: distilled water) from days 5 to 29 of gestation. The animals were examined on day 30 of gestation. At 405 mg/kg body weight and day, 1 dam died and at 1215 mg/kg body weight and day, 2 dams died. Vaginal bleeding and lethargy occurred in another dam in the 405 mg/kg group, which is why the animal was sacrificed. Two dams in the 1215 mg/kg group had a spontaneous abortion or premature birth. At this dose, 3 dams had diarrhoea. At and above 405 mg/kg body weight and day, the body weight gains in the dams (days 20 to 23 of gestation) were reduced. There was a statistically significant reduction in foetal weights in the high dose group (42.24 g ± 8.77 g, controls: 44.36 g ± 6.58 g; 5%). No other developmental toxic effects, including teratogenicity, were observed. The NOAEL for developmental toxicity was 405 mg/kg body weight and day and the NOAEL for maternal toxicity was 135 mg/kg body weight and day (JRF 2005).

A NOAEL for perinatal toxicity of 1000 mg TMAD/kg body weight and day, the highest dose tested, was derived from the 2-generation study in rats described in Section 5.5.1 (NOTOX B.V. 2007 a).

### 5.6 Genotoxicity

#### 5.6.1 In vitro

In bacterial mutagenicity tests with the Salmonella strains TA98, TA100, TA1535, TA1537 with and without the addition of a metabolic activation system, TMAD did not increase the number of revertants in the standard plate incorporation test with 0, 40, 200, 1000, 5000 or 10 000 µg/plate and in the standard plate incorporation test and preincubation test with 0, 250, 500, 1000, 2000 or 3000 µg/plate. The substance was therefore not mutagenic. Cytotoxicity occurred at and above 5000 µg/plate and at and above 2000 µg/plate, respectively (BASF AG 1995; ECHA 2018).

Chromosomal aberration tests in V79 cells (Chinese hamster lung fibroblasts) according to OECD Test Guideline 473, with and without the addition of a metabolic activation system (S9 mix from rat liver, inducers: phenobarbital/beta-naphthoflavone), were carried out with 0 or 10–100 µg TMAD/ml (dissolved in deionized water). Incubation was carried out for 4 hours. A statistically significant increase in the frequencies of chromosomal aberrations occurred with and without metabolic activation at 40 µg/ml and above. Cytotoxic effects were seen both without and with metabolic activation at 60 µg/ml and above (ECHA 2018; RCC 2002 b). The substance is therefore to be regarded as clastogenic in vitro.

Two HPRT gene mutation tests with V79 cells (Chinese hamster lung fibroblasts) carried out according to OECD Test Guideline 476 with and without the addition of a metabolic activation system (S9 mix) did not reveal an increase in mutations at TMAD concentrations of 10–80 µg/ml. Cytotoxicity was observed at 60 µg/ml and above (ECHA 2018; RCC 2002 a).

#### 5.6.2 In vivo

Single gavage doses of 0, 1000 or 2000 mg TMAD/kg body weight in 4 male Wistar/Han rats did not lead to increased DNA repair synthesis after 2 or 16 hours in the UDS test (RCC 2002 c).

In accordance with OECD Test Guideline 474, groups of 5 male and 5 female NMRI mice were given single intraperitoneal injections of 0, 375, 750 or 1500 or 0, 437.5, 750 or 1750 mg TMAD/kg body weight (dissolved in deionized water). The micronuclei of the polychromatic erythrocytes (PCE) in the bone marrow were examined after 24 hours and additionally after 48 hours in the animals treated with the high dose. The incidence of micronuclei was increased slightly, but not in a statistically significant manner (p = 0.09) in the male animals at 750 mg/kg body weight and above. At the high doses, there was an increase in normochromatic erythrocytes (NCE) (as a ratio of PCE/NCE) and thus a cytotoxic effect on the bone marrow (ECHA 2018; RCC 2002 d, 2003).

### 5.7 Carcinogenicity

There are no studies with TMAD available. However, since TMAD spontaneously releases formaldehyde in aqueous solution, which has a local carcinogenic effect (Greim 2002), also TMAD can be expected to cause local carcinogenicity.

## 6 Manifesto (MAK value/classification)

The critical effects are the effects on the larynx, the carcinogenicity of the hydrolysis product formaldehyde and the sensitizing effects on the skin.

**MAK value. **Tetramethylol acetylenediurea hydrolyses rapidly in aqueous solution to form formaldehyde, which causes significant irritation in the nose and is carcinogenic to the epithelium of the nasal mucosa after chronic exposure (Greim 2002; Hartwig 2014).

A MAK value for formaldehyde releasers is derived, in analogy to that for formaldehyde, dependent on the vapour pressure (no aerosol formation), the rate of hydrolysis and the number of formaldehyde molecules released per molecule of the substance to be assessed.

The half-life of the hydrolysis rate of TMAD at pH 7 is about 0.9 hours at 30 °C. The calculated vapour pressure at 20 °C is so low that exposure to aerosol rather than vapour must be assumed. In studies carried out according to OECD test guidelines, TMAD was slightly irritating to the skin and not irritating to the eye.

The effects on the larynx observed at the lowest concentration tested of 21.7 mg/m^3^ in the 28-day inhalation study (TNO 2005) indicate an effect of the aerosol used. The effects in the nose to be expected after hydrolytic release of the formaldehyde became apparent only at the next-higher concentration. Since the substance was used as an aqueous solution, the effects are probably due to the TMAD aerosol and the effects of the formaldehyde.

Squamous metaplasia of the epiglottis of low severity at the entrance to the larynx is not to be regarded as adverse (Kaufmann et al. 2009). At the lowest concentration tested of 21.7 mg/m^3^, metaplasia of low severity occurred in the larynx. However, 1 female animal displayed hyperplasia of the larynx at this concentration. In 1 male animal, metaplasia was not observed in the epiglottis, but in the lateral wall of the larynx. In addition, the concentration–response relationship for the changes in the larynx is steep. Thus, the lowest concentration tested is to be regarded as the LOAEC. For slight, incipient effects at the LOAEC, the Commission extrapolates with a factor of 3 to the NAEC (no adverse effect concentration). Hyperplasia occurred in the nasal cavity at 96 mg/m^3^ and above.

Based on the NAEC, local effects on the upper respiratory tract due to formaldehyde are extrapolated according to the method of Brüning et al. (2014) from subacute to chronic exposure (1:2) and from animals to humans (1:3). For the impaction of the substance in the larynx, the increased respiratory volume at the workplace (1:2) is taken into consideration, resulting in a concentration of 0.6 mg/m^3^. Using the preferred value approach, a MAK value of 0.5 mg TMAD/m^3^ I (inhalable fraction) is established.

When used in diluted aqueous solutions, complete hydrolysis should be expected and therefore the MAK value for formaldehyde (Greim 2002; Hartwig 2014) should be observed.

Hardly any data are available for the hydrolysis product acetylenediurea. The substance was not irritating to the eyes and skin and not toxic up to 1000 mg/kg body weight and day in a 28-day study with oral administration (ECHA 2019). Acetylenediurea was not genotoxic in the in vivo micronucleus test in the male mouse and in the bacterial Salmonella mutagenicity test with TA100 (NTP 2018 a, b).

**Peak limitation. **As the critical effect is irritation, TMAD has been assigned to Peak Limitation Category I with an excursion factor of 2, in analogy to the classification made for formaldehyde.

**Prenatal toxicity. **A prenatal developmental toxicity study with gavage doses of TMAD of 0, 135, 405 or 1215 mg/kg body weight and day in New Zealand White rabbits revealed increased mortality, diarrhoea and reduced body weights at and above 405 mg/kg body weight and day. The reduction in the foetal weights was of statistical significance at 1215 mg/kg body weight and day. The NOAEL (no observed adverse effect level) for developmental toxicity was 405 mg TMAD/kg body weight and day and the NOAEL for maternal toxicity 135 mg TMAD/kg body weight and day (JRF 2005). A NOAEL for perinatal and parental toxicity of 1000 mg TMAD/kg body weight and day, the highest dose tested, was derived from the 2-generation study in Wistar rats (NOTOX B.V. 2007 a).

The following toxicokinetic data are taken into consideration for the extrapolation of the NOAELs for prenatal and perinatal developmental toxicity of 405 mg/kg body weight and day in rabbits and 1000 mg/kg body weight and day in rats to a concentration in workplace air: the daily exposure of the animals in comparison with the 5 days per week exposure at the workplace (7:5) (in the study carried out according to OECD Test Guideline 416), the corresponding species-specific correction values for the rat and the rabbit (1:4 and 1:2.4), the oral absorption (22%; ECHA 2018; NICNAS 2016), the body weight (70 kg) and respiratory volume (10 m^3^) of the person, and the assumed 100% absorption by inhalation.

The concentrations calculated from this are 260 and 539 mg/m^3^, which correspond to 520 and 1078 times the MAK value of 0.5 mg/m^3^, respectively. As the margin to the MAK value is sufficiently large and no teratogenicity was found, TMAD has been assigned to Pregnancy Risk Group C.

**Carcinogenicity. **Tetramethylol acetylenediurea hydrolyses rapidly in aqueous solution to form formaldehyde, which can cause significant irritation in the nose and, with chronic exposure, tumours in the epithelium of the nasal mucosa.

Since the release of formaldehyde at the nasal mucosa cannot be excluded, TMAD has been classified in Carcinogen Category 4. A maximum of 46% formaldehyde (0.23 mg/m^3^) is produced during exposure at the level of the MAK value for TMAD. This concentration is below the MAK value for formaldehyde (0.37 mg/m^3^). Therefore, TMAD is not expected to contribute to the risk of cancer in humans if the MAK value is observed.

**Germ cell mutagenicity. **Tetramethylol acetylenediurea is clastogenic in vitro, which has not been confirmed in vivo. Mutagenicity was not observed in vitro. Studies investigating germ cells are not available.

The hydrolysis product formaldehyde is classified in Category 5 for germ cell mutagens. This means that if the MAK value of 0.3 ml formaldehyde/m^3^ is observed, only a very small contribution to the genetic risk for humans is to be expected.

Since only a very small contribution to germ cell mutagenicity is to be expected if the MAK value for TMAD is observed, TMAD has been classified in Category 5 for germ cell mutagens.

**Absorption through the skin. **There are no data available for the absorption of TMAD through the skin in humans, but experimental data are available from a study in rats with dermal exposure to a 3% solution for 6 hours. The dermal LD_50_ value for acute exposure in rats is given as more than 2000 mg/kg body weight. No reliable data are available for repeated dermal exposure. Even at the lowest dose tested of 100 mg/kg body weight, the substance caused severe local irritation in all animals after application for 28 days.

From the dermal penetration study in vivo with a 3% solution, a maximum systemic exposure of 0.475 mg or 6.8 µg/kg body weight for a person of 70 kg body weight can be estimated under standard conditions (2000 cm^2^ of skin, exposure for 1 hour) (Section 3.1). This is several orders of magnitude below the NOAEL values from the 28-day and 90-day oral feeding studies in rats (1000 mg/kg body weight, no substance-related systemic effects observed) and a developmental toxicity study in rabbits (NOAEL 125 mg/kg body weight; end point increased mortality and reduced body weights). Therefore, TMAD has not been designated with an “H” (for substances which can be absorbed through the skin in toxicologically relevant amounts).

This likewise applies in view of the possible rapid systemic release of formaldehyde in the bloodstream: for a 70 kg person, the maximum total amount of TMAD absorbed under the conditions mentioned is 0.475 mg (equivalent to 0.0018 mmol). Assuming rapid complete hydrolysis, this results in the release of 0.007 mmol formaldehyde (0.22 mg). The physiological formaldehyde level in human blood is about 2–3 mg/l or 10–15 mg in 5 litres of blood (AGS 2022; Heck et al. 1985). The additional maximum contribution of 0.22 mg formaldehyde does not exceed 10% of the physiological formaldehyde range and thus lies within the variation range of background exposure. It is therefore not designated with an “H” (for substances which can be absorbed through the skin in toxicologically relevant amounts).

**Sensitization. **A positive result from a maximization test with TMAD provides evidence of skin sensitization. In addition, there are clinical findings that substantiate the skin-sensitizing effect. All in all, and due to its ability to release formaldehyde, the substance has been designated with “Sh” (for substances which cause sensitization of the skin). Data for sensitization of the respiratory tract are not available. The substance has therefore not been designated with “Sa” (for substances which cause sensitization of the airways).
